# Impact of the Japanese 5S management method on patients’ and caretakers’ satisfaction: a quasi-experimental study in Senegal

**DOI:** 10.3402/gha.v9.32852

**Published:** 2016-11-28

**Authors:** Shogo Kanamori, Marcia C. Castro, Seydou Sow, Rui Matsuno, Alioune Cissokho, Masamine Jimba

**Affiliations:** 1 Department of Community and Global Health, Graduate School of Medicine, The University of Tokyo, Tokyo, Japan; 2 IC Net Limited, Saitama, Japan; 3 Department of Global Health and Population, Harvard T. H. Chan School of Public Health, Boston, MA, USA; 4 Agence Africaine de Santé Publique, Dakar, Senegal; 5 Faculty of Medicine School of Health Sciences, Gunma University, Maebashi, Japan; 6 Actions pour le Changement, Dakar, Senegal

**Keywords:** 5S, quality of healthcare, program evaluation, patient satisfaction, Senegal

## Abstract

**Background:**

The 5S method is a lean management tool for workplace organization, with 5S being an abbreviation for five Japanese words that translate to English as Sort, Set in Order, Shine, Standardize, and Sustain. In Senegal, the 5S intervention program was implemented in 10 health centers in two regions between 2011 and 2014.

**Objective:**

To identify the impact of the 5S intervention program on the satisfaction of clients (patients and caretakers) who visited the health centers.

**Design:**

A standardized 5S intervention protocol was implemented in the health centers using a quasi-experimental separate pre-post samples design (four intervention and three control health facilities). A questionnaire with 10 five-point Likert items was used to measure client satisfaction. Linear regression analysis was conducted to identify the intervention's effect on the client satisfaction scores, represented by an equally weighted average of the 10 Likert items (Cronbach's alpha=0.83). Additional regression analyses were conducted to identify the intervention's effect on the scores of each Likert item.

**Results:**

Backward stepwise linear regression (*n=*1,928) indicated a statistically significant effect of the 5S intervention, represented by an increase of 0.19 points in the client satisfaction scores in the intervention group, 6 to 8 months after the intervention (*p=*0.014). Additional regression analyses showed significant score increases of 0.44 (*p=*0.002), 0.14 (*p=*0.002), 0.06 (*p=*0.019), and 0.17 (*p=*0.044) points on four items, which, respectively were healthcare staff members’ communication, explanations about illnesses or cases, and consultation duration, and clients’ overall satisfaction.

**Conclusions:**

The 5S has the potential to improve client satisfaction at resource-poor health facilities and could therefore be recommended as a strategic option for improving the quality of healthcare service in low- and middle-income countries. To explore more effective intervention modalities, further studies need to address the mechanisms by which 5S leads to attitude changes in healthcare staff.

## Introduction

The term 5S is an abbreviation for five Japanese words, *Seiri*, *Seiton*, *Seisou*, *Seiketsu*, and *Shitsuke*, which largely pertain to maintaining cleanliness. These five words translate to English as Sort, Set in Order, Shine, Standardize, and Sustain, respectively, and characterize a set of practices designed to improve workplace organization and productivity (1–4). The 5S management method (hereinafter abbreviated as ‘5S’) is known as the foundation of the lean approaches, which maximize value by removing wasteful factors (5). It evolved in the Japanese manufacturing sector and was introduced to the West in the 1980s (2). Currently, 5S is used in healthcare settings to organize and standardize the workplace for lean healthcare (6). Because of its low cost and technologically undemanding features, 5S is considered an appropriate starting point for improving healthcare services (3, 6–9).

The impact of 5S, often combined with other lean methods, has been researched in the health sectors of the United States (10–13), India (14), Jordan (15), Sri Lanka (16), and Senegal (17). However, little is known about its impact on healthcare-service users. In addition, the feasibility of implementing 5S has not been established in resource-poor settings, although a few studies have already targeted health facilities in low- and middle-income countries (LMICs) (18).

Although evidence is limited, 5S has been suggested as a method to improve the quality of government healthcare services, particularly in LMICs (19). In Sri Lanka, the Castle Street Hospital for Women, a public hospital, first documented its implementation results (16). Its success led to widespread 5S implementation and eventually the Sri Lankan government adopted it as a mainstream strategy to improve the quality of government healthcare services (20). Aiming to replicate Sri Lanka's positive experience, the Asia–Africa Knowledge Cocreation Program (AAKCP) of the Japan International Cooperation Agency (JICA) introduced 5S to selected government hospitals in 15 African countries – Benin, Burkina Faso, Burundi, the Democratic Republic of Congo, Eritrea, Kenya, Madagascar, Malawi, Mali, Morocco, Niger, Nigeria, Senegal, Tanzania, and Uganda – between 2007 and 2013 (19, 21). Tanzania's Health Ministry adopted 5S as part of the government's strategy to improve the quality of its healthcare services (22).

In Senegal, the Ministry of Health and Social Action (Ministère de la Santé et de l'Action Sociale; MSAS) conducted a project titled ‘Project for the Reinforcement of the Health System in Senegal (Projet d'Appui au Renforcement du Système de Santé au Sénégal; PARSS)’, with technical support from JICA. As a technical cooperation project aimed to strengthen the health systems in the Tambacounda and Kedougou regions (out of 14 regions in the country), PARSS was designed primarily with activities to address several strategic domains. One of these activities focused on introducing 5S to health centers in the two regions, using knowledge gained from the AAKCP pilot initiative. However, the project also comprised research activities to measure the 5S intervention's impact. The project was conducted between April 2011 and March 2014. The ultimate objective of the PARSS was to establish a sustainable and replicable mechanism for introducing 5S to health centers based on prior experience in the Tambacounda and Kedougou regions.

This study was conducted to identify the impact of the 5S intervention at health centers in the Tambacounda and Kedougou regions on the satisfaction levels of patients and caretakers visiting these health centers. In our study, the term ‘impact’ was used in the context of the impact assessment that has been defined by Vanclay and Bronstein as ‘prediction or estimation of the consequences of a current or proposed action’ (23).

## Methods

### Target areas and facilities

The Tambacounda and Kedougou regions, located in the eastern part of Senegal, are characterized by comparatively lower healthcare services and economic indicators than other parts of Senegal; for example, 79% of the women in Tambacounda and 82% in Kedougou received antenatal care from skilled providers, whereas the country's average was 93.3%. In addition, 52.9% of the population in Tambacounda and 61.3% in Kedougou fell within the first wealth quintile (i.e. the poorest 20% in the country's population) (24).

The Tambacounda and Kedougou regions are partitioned into 10 Health Districts: 7 (Bakel, Dianke Makha, Goudiry, Kidira, Koumpentoum, Makacolibanta, and Tambacounda) in Tambacounda; and 3 (Kedougou, Salemata, and Saraya) in Kedougou. Under the Senegal government's health system, health districts provide primary healthcare through health centers and health posts (25). The health centers are secondary-level facilities headed by a chief medical doctor and are organized around services or units, including medical consultation, gynecology or obstetrics care, operation rooms, dental care, emergency care (medical and surgical), radiology, laboratory, pharmacy, and inpatient wards (medical, surgical, and gynecology or obstetric) (26). Health posts, headed by a chief nurse, are the primary-level facilities that provide primary healthcare services with limited inpatient facilities (25). In Tambacounda and Kedougou, each of the 10 health districts has one health center located at its center and several health posts dotted around the periphery. The number of staff members at each health center ranged between 16 and 78, as identified by the survey team (no official records of the number of staff members existed before our study).

### Overall design of PARSS

**Fig. 1:**
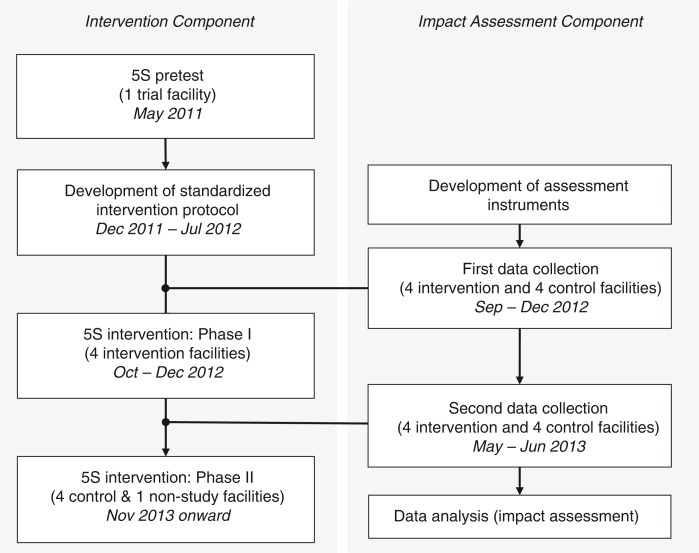
Overall design of PARSS with intervention and impact assessment components.

The PARSS design included two components: 1) the intervention component to introduce 5S to the health centers and 2) the impact assessment component to identify the intervention's impact (Fig. 1). The activities involved in both components were scheduled to allow a quasi-experimental study to be conducted between March 2011 and February 2014. Ten health centers in the two regions, considered pilot facilities under PARSS, were divided into three groups: 1) one trial facility, 2) eight study facilities (four intervention and four control facilities), and 3) one non-study facility. At the kick-off meeting for PARSS, conducted in Tambacounda in May 2011, the Tambacounda Health Center, located at the center of the Tambacounda region, was selected as the trial facility to pretest the 5S intervention primarily because of its physical accessibility to the project stakeholders. The Salemata Health Center in the Kedougou region was designated a non-study facility because its service capacity was significantly lower and because it had been only nominally upgraded from a health post to a health center in 2010, with little upgradation to its infrastructure or workforce. Therefore, it was considered unsuitable as a study facility.

Among the eight study facilities, four health centers (Bakel, Dianke Makha, Goudiry, and Kedougou) were assigned to the intervention group and the other four (Kidira, Koumpentoum, Makacolibantang, and Saraya) to the control group. The allocation of the facilities to the intervention and control groups was based on practical reasons rather than random selection. New building facilities were constructed in Kidira, Koumpentoum, Makacolibantang, and Saraya; thus, these health centers were expected to move at any time to the new locations. However, it was unlikely that the relocations would be completed in time for the 5S intervention (Phase I) planned for the latter part of 2012 because electricity and water systems had not been installed. The project stakeholders agreed not to initiate the 5S intervention at those health centers until they had relocated to the new facilities; therefore, those four health centers were allocated to the control group. It was envisioned that the first and second data collections would be conducted at the old facilities and the 5S intervention (Phase II) would be conducted at the new facilities after mid-2013. The remaining four health centers without relocation plans were allocated to the intervention group.

### Intervention component

#### Pretest of the 5S intervention at the trial facility

The 5S intervention was pretested at the Tambacounda Health Center in May 2011. The objectives of the pretest included the following aspects: 1) establishing a reference health center to showcase the 5S method, 2) testing and validating the 5S training instruments, and 3) obtaining preliminary insight into a viable intervention protocol that would be standardized and subsequently used in the eight study facilities. The activities conducted in connection with this pretest are included in a qualitative research study conducted at the Tambacounda Health Center (17).

#### Development of a standardized 5S intervention protocol

Based on the experience gained through the 5S pretest at the Tambacounda Health Center, PARSS conducted a series of activities to identify a viable protocol and standardized guidelines for conducting the 5S intervention. All of the activities were conducted in a participatory manner. Between December 2011 and July 2012, the project's stakeholders met several times to draft guidelines pertaining to the 5S intervention protocol at the health centers. A standardized organizational structure was proposed in the first draft of the guidelines and the 5S Program was defined as a 5-day intervention package, consisting of workshops and practice sessions (Table 1) (27). The guidelines proposed that the 5S intervention team was to be responsible for facilitating and coaching the 5S Program and that the program was to be composed of one or two supervisor(s), four qualified mentors, and four apprentice mentors. Apprentice mentors were to be nominated from among the health center staff members where the 5S Program was to be implemented. They were expected to become qualified mentors after experiencing the program in a learning-by-doing system, and to assist in its implementation in other health centers. The guidelines detailed all other arrangements, including standardized daily proceedings, the content of the workshop and practice sessions, and job aids to practice and monitor 5S.

**Table 1:** Standardized organization of the 5S Program at the health centers according to the guidelines

Duration and Schedule	Five days (Monday to Friday, or Tuesday to Saturday), composed of:
	– Day 1: Workshop Session (Orientation and Introductory Lectures)
	– Days 2&3: On-site 5S Practice
	– Days 4&5: Workshop Session (Evaluation and Wrap-up)
Composition of 5S Intervention Team	Nine or ten persons, including:
	– One or two supervisors (from MSAS or Medical Region office)
	– Four qualified mentors (external personnel of the health center; formerly served as apprentice mentors)
	– Four apprentice mentors (staff members of the health center)
Participants	All the staff members of the health center, including clinical, administrative, and support staff.

In July 2012, the PARSS members conducted the 5S Program at Gaspard Kamara Health Center, located in the capital city of Dakar. This activity had two objectives: 1) to test and validate the protocol of the 5S Program prescribed in the draft guidelines and 2) to foster a new cadre of mentors who could serve as qualified mentors during the 5S intervention at any of the health centers, including those in the Tambacounda and Kedougou regions. After adjusting the content, such as the time allocation for each activity and the procedures for the on-site practice of 5S, the draft guidelines were updated to a version for validation and finalization during field applications in the two regions.

#### 5S intervention in the target regions

The 5S intervention was conducted at health centers in the Tambacounda and Kedougou regions in accordance with the design of PARSS's impact assessment component (Fig. 1). During Phase I of the intervention period, between October and December 2012, the 5S Program was conducted at four intervention facilities (Bakel, Dianke Makha, Goudiry, and Kedougou), in accordance with the protocol guidelines. The PARSS members finalized the document by incorporating experience related to the intervention process in the four facilities, as well as the project stakeholders’ opinions. The Senegalese government adopted it as the official guidelines in July 2013 (27). Subsequently, Phase II of the intervention period involved two health centers (Makacolibantang and Salemata) between November and December 2013. The 5S intervention was not feasible at the remaining three health centers because the relocations to the new facilities were not realized by the end of the project period.

The coordinator of MSAS's National Quality Program (Programme National Qualité; PNQ) managed the 5S intervention process in the two regions. This person also shared the supervisor's role in the 5S intervention teams with other members of MSAS and the two medical regions. Apprentice mentors were fostered as qualified mentors through the 5S intervention at the health centers. In the end, 34 people in the two regions became qualified mentors.

### Impact assessment component

#### Development of the assessment instruments

The assessment focused on the 5S intervention's impact on the satisfaction levels of patients and caretakers who visited the health centers (henceforth, ‘client satisfaction’). A questionnaire was developed to measure their satisfaction from the time they entered the health center to the time of exit. Questions were designed to elicit information about how patients and caretakers perceived the services provided while waiting for and receiving them, and upon completing the entire process. The respondents were to answer all of the questions on a five-point Likert scale, which ranged from ‘strongly agree’ to ‘strongly disagree’. Other questions were also added to assess respondents’ experience related to their visit (e.g. service unit visited, means of transportation used to visit the facility, whether they paid for the service), their demographic and socio-economic characteristics (e.g. age, sex, marital status), and their household possessions (used to construct an asset index). To ensure clarity and to gain preliminary insight into the questions’ validity, the questionnaire was pilot-tested with patients and caretakers at the Tambacounda Health Center and two health centers in Dakar, including the Gaspard Kamara Health Center. Through this testing process, the questionnaire was refined to include 10 items measuring client satisfaction (Table 4).

#### Sampling and data collection

The questionnaire was administered twice at the eight study facilities, that is, before and after Phase I of the 5S intervention (Fig. 1). The first data collection was conducted between September and December 2012. This period overlapped with Phase I of the 5S intervention (October–December 2012); however, the two activities (intervention and data collection) were scheduled in a sequence, such that the data collection always preceded the 5S intervention in the intervention facilities. The second data collection was conducted between May and June 2013, approximately 6 to 8 months after completing the Phase I intervention.

Interviewees were patients or caretakers who had experienced clinical services at the following seven service units of the health centers: 1) maternity, 2) outpatient medical clinic, 3) outpatient nurse clinic, 4) prenatal care, 5) inpatient wards, 6) family planning, and 7) immunization clinic. A convenience sampling method was used. Trained interviewers conducted face-to-face individual interviews in local languages with patients or caretakers exiting from the service units. Their refusal to participate was almost nil; only one participant was unwilling and declined to answer questions about socio-economic status. The participants did not receive any financial incentives for their participation in the interviews. The total numbers of participants at the eight health centers reached 1,300 and 1,087 during the first and the second data collection stages, respectively (Table 2). Five consecutive operating days were devoted to data collection at each of the eight health centers at each stage.

**Table 2:** Numbers of the study participants by data collection stage at the eight study facilities (*n=*2,387)

	Control Group^a^					Intervention Group^a^					
			
Data Collection Stage	KI	KO^b^	MA	SA	Sub-Total	BA	DI	GO	KE	Sub-Total	Total
First data collection stage	159	168	192	89	608	199	114	199	180	692	1,300
Second data collection stage	107	190	108	128	533	170	91	106	187	554	1,087

a The names of the health centers are abbreviated as: KI: Kidira; KO: Koumpentoum; MA: Makacolibantang; SA: Saraya; BA: Bakel; DI: Dianké Makha; GO: Goudiry; and KE: Kédougou.

b The data collected at KO (*n=*358) were excluded from the dataset used for the analysis.

#### Assessment of the reliability and validity of the client satisfaction questionnaire

To assess the reliability and validity of the 10-item scale measuring client satisfaction, the data from the first data collection stage were used (*n=*1,204). Based on the scores on the Likert items (strongly agree=5, agree=4, neutral=3, disagree=2, and strongly disagree=1), the internal consistency reliability of the client satisfaction scale was measured using Cronbach's alpha (28). Factor analysis was conducted to assess its construct validity.

Cronbach's alpha for the 10-item scale measuring client satisfaction was 0.83, which indicated the scale's high reliability. Factor analysis showed that the eigenvalue of the principal factor was 3.51, with a difference of 3.17 between it and the second factor, indicating that the 10 items represented a single construct pertaining to client satisfaction. Based on these results, it was considered appropriate to use this 10-item scale as the outcome measurement tool.

#### Data analysis (impact assessment)

The intervention's effect on client satisfaction was analyzed using a separate pre-post samples design. The data collected at the Koumpentoum Health Center during the first and the second data collection stages (*n=*358) were excluded from the original dataset (*n=*2,387) after it was found that the outpatient medical clinic provided free-of-charge services at the time of the second data collection. The reasons for this were not known. This practice, which was unique to the Koumpentoum Health Center, was considered a potential confounder. As it was impossible to adjust for this factor in the statistical model, only data from the seven health facilities (three control and four intervention facilities) were used for the analyses. The following groups were also excluded from the data analysis: patients under 18 years of age who were not accompanied by a caretaker (*n=*34), caretaker respondents under 18 years of age (*n=*20), caretaker respondents who were not with the patient while at the health facility (*n=*25), caretaker respondents not living in the same house as the patient (*n=*20), those visiting the health center for reasons other than medical concerns (*n=*1), and those who chose not to complete the interview (*n=*1). The final sample size used for the analysis was 1,928.

A ‘client satisfaction score’ (a continuous variable) was defined as an equally weighted average score of the 10 Likert items. A linear regression analysis was conducted to identify the effect of the 5S intervention on the client satisfaction scores, using the following covariates: ‘intervention’ (control=0 and intervention=1) and ‘data collection stage’ (first stage=0 and second stage=1). The regression model also included an interaction term of intervention by data collection stage (intervention×data collection stage), which is the principal outcome variable to assess the intervention's effect using a separate pre-post samples design. Respondents’ demographic and socio-economic characteristics, including an asset index, were used to control for possible confounding. The asset index was calculated based on a principal component analysis of the respondents’ household possessions (vehicle or motorcycle, TV, refrigerator or freezer, radio, landline telephone, mobile phone, bicycle, fan, clock or watch, sofa, wall material of the house, floor material of the house, source of potable water, source of electricity, and toilet in the house) (29). Three different regression models were examined: 1) without adjusting for the control variables, 2) adjusting for all of the possible control variables, and 3) adjusting for the control variables selected by backward stepwise regression using *p=*0.15 entry and *p=*0.05 removal criteria. Because health facilities were not randomly assigned to the control and intervention groups, fixed effects were used for the facilities in each group.

Additionally, linear regression analyses were conducted to identify the intervention's effect on the scores of each of the 10 Likert-scale question items. Using the same variables as those in the aforementioned regression analysis, 10 separate analyses were conducted using backward stepwise regression (*p=*0.15 entry and *p=*0.05 removal).

### Ethical considerations

This study was approved by the National Ethical Committee for Medical Research of MSAS in Senegal (No. 1261) and the Research Ethics Committee of the University of Tokyo in Japan (No. 3781). Participation in the study was voluntary. Each participant provided informed consent in writing before each interview. Participants were informed that they could withdraw from the interviews at any time without any risk of sanctions. To guarantee privacy, the interviews were conducted in a place where conversations were not audible to other people. Participants were not asked to reveal their names during the interviews, and they were assured that their anonymity would be protected throughout the data collection and analyses procedures.

## Results

The characteristics of the respondents included in the analyses (*n=*1,928) are presented in Table 3. At the first data collection stage, the sample sizes of the study participants from the control facilities (Group A) and the intervention facilities (Group B) were 410 and 640, respectively. At the second data collection stage, the sample sizes were 330 and 540 from the control facilities (Group C) and the intervention facilities (Group D), respectively. The proportions of the patient respondents in Group A, B, C, and D were 59.5, 50.9, 52.1, and 65.4%, respectively.

**Table 3:** Characteristics of the study participants included in the analysis (*n=*1,928)

	First data collection stage		Second data collection stage	
		
	Control facilities	Intervention facilities	Control facilities	Intervention facilities
	
	*n=*410 (Group A)	*n=*640 (Group B)	*n=*338 (Group C)	*n=*540 (Group D)
Health center				
Kidira	148	–	105	–
Koumpentoum	(Excluded)	–	(Excluded)	–
Makacolibantang	182	–	105	–
Saraya	80	–	128	–
Bakel	–	186	–	169
Dianké Makha	–	105	–	90
Goudiry	–	180	–	106
Kédougou	–	169	–	175
Respondent	(%)	(%)	(%)	(%)
Patient	59.5	50.9	52.1	65.4
Caretaker	40.5	49.1	47.9	34.6
Sex of respondent				
Male	24.6	26.1	34.6	23.7
Female	75.1	73.9	65.1	76.1
Missing	0.2	0.0	0.3	0.2
Age of respondent				
18 to <25 years	32.9	36.4	37.0	37.4
25 to <35 years	36.8	35.6	41.4	35.2
35 to <45 years	14.6	16.3	14.2	13.2
≥45 years	12.7	11.7	7.4	14.3
Service unit visited				
Maternity	8.3	6.1	5.9	13.7
Outpatient medical clinic	4.9	9.7	7.1	6.5
Outpatient nurse clinic	43.4	36.3	38.8	39.4
Prenatal care	16.6	18.3	18.9	20.6
Inpatient ward	6.6	9.1	10.7	6.1
Family planning	12.9	4.2	2.4	5.6
Immunization	7.3	16.4	16.3	8.2
Means of transportation used to visit the facility				
Personal vehicle or motorbike	21.2	26.4	34.3	29.4
Public transport	24.9	36.7	14.5	34.4
By walk or bicycle	53.9	36.9	51.2	36.1
Visiting the facility first time	9.5	14.5	19.2	17.6
Paid money for the service	81.5	89.1	92.6	88.3
How long living in the current residence?				
<5 years	18.8	23.0	24.3	26.1
5 to <10 years	9.8	10.9	9.8	11.9
10 to <20 years	22.4	21.1	20.7	17.8
20 to <30 years	23.2	25.5	23.4	21.5
≥30 years	25.9	19.5	21.9	22.8
Marital status				
Married	84.9	84.8	85.2	89.6
Single	10.0	10.0	12.1	8.3
Divorced	1.7	2.2	1.2	0.7
Widowed	3.4	3.0	1.5	1.3
Any formal education attended	30.5	38.3	40.2	36.7
Occupation of household head				
Informal sector	70.0	59.7	80.8	70.6
Formal sector	14.2	20.3	13.9	14.4
Working abroad	9.0	12.2	4.7	12.6
Not working	2.4	5.5	0.0	1.3
Other	4.4	2.3	0.6	1.1

Table 4 shows the mean scores and frequencies of the participants’ responses to each of the 10 Likert-scale question items for Groups A to D. The mean client satisfaction score (i.e. the mean of the individual respondent's 10-item mean scores) was 4.01 (SD 0.51) for the control facilities (Group A) and 3.98 (SD 0.59) for the intervention facilities (Group B) at the first data collection stage. It was 4.07 (SD 0.51) for the control (Group C) and 4.20 (SD 0.59) for the intervention group (Group D) at the second data collection stage. Seven of the 10 items showed greater mean scores for the control facilities at the second data collection phase (Group C) than those at the first data collection phase (Group A). Meanwhile, the scores were greater in all 10 items for the intervention facilities at the second data collection phase (Group D) than those at the first data collection phase (Group B).

**Table 4:** Mean scores and frequencies of the participants’ responses to the 10-item Likert-scale questionnaire measuring client satisfaction (*n=*1,928)

		First data collection phase		Second data collection phase	
			
Question Items		Control facilities *n=*410 (Group A)	Intervention facilities *n=*640 (Group B)	Control facilities *n=*338 (Group C)	Intervention facilities *n=*540 (Group D)
*Individual items*^a^					
1. The duration of your consultation by healthcare staff was appropriate.					
Mean scores (SD)		4.01 (0.84)	3.98 (0.91)	3.87 (0.73)	4.05 (0.84)
Frequencies of responses (%)	1	10 (2.4)	11 (1.7)	2 (0.6)	4 (0.7)
	2	5 (1.2)	36 (5.6)	18 (5.3)	30 (5.6)
	3	68 (16.6)	94 (14.7)	48 (14.2)	64 (11.9)
	4	215 (52.4)	312 (48.8)	223 (66.0)	277 (51.3)
	5	112 (27.3)	187 (29.2)	47 (13.9)	165 (30.6)
	Missing	0 (0.0)	0 (0.0)	0 (0.0)	0 (0.0)
2. The explanation about your illness/case and the medication(s) provided by the healthcare staff during the consultation were appropriate.					
Mean scores (SD)		4.29 (0.75)	4.17 (0.83)	4.16 (0.68)	4.33 (0.76)
Frequencies of responses (%)	1	3 (0.7)	10 (1.6)	2 (0.6)	4 (0.7)
	2	7 (1.7)	17 (2.7)	5 (1.5)	9 (1.7)
	3	33 (8.0)	64 (10.0)	25 (7.4)	37 (6.9)
	4	190 (46.3)	306 (47.8)	196 (58.0)	211 (39.1)
	5	172 (42.0)	236 (36.9)	93 (27.5)	229 (42.4)
	Missing	5 (1.2)	7 (1.1)	17 (5.0)	50 (9.3)
3. The waiting time before you received the consultation was within a reasonable timeframe.					
Mean scores (SD)		3.57 (1.07)	3.48 (1.15)	3.45 (0.98)	3.56 (1.17)
Frequencies of responses (%)	1	23 (5.6)	43 (6.7)	11 (3.3)	36 (6.7)
	2	38 (9.3)	94 (14.7)	57 (16.9)	79 (14.6)
	3	109 (26.6)	131 (20.5)	69 (20.4)	86 (15.9)
	4	163 (39.8)	255 (39.8)	172 (50.9)	225 (41.7)
	5	77 (18.8)	116 (18.1)	29 (8.6)	113 (20.9)
	Missing	0 (0.0)	1 (0.2)	0 (0.0)	1 (0.2)
4. The professional competence of the healthcare staff at this health facility is high.					
Mean score (SD)		4.16 (0.72)	4.16 (0.88)	4.27 (0.84)	4.43 (0.88)
Frequencies of responses (%)	1	2 (0.5)	9 (1.4)	2 (0.6)	8 (1.5)
	2	4 (1.0)	16 (2.5)	10 (3.0)	13 (2.4)
	3	52 (12.7)	97 (15.2)	43 (12.7)	49 (9.1)
	4	215 (52.4)	250 (39.1)	120 (35.5)	127 (23.5)
	5	131 (32.0)	255 (39.8)	159 (47.0)	326 (60.4)
	Missing	6 (1.5)	13 (2.0)	4 (1.2)	17 (3.1)
5. The way the healthcare staff communicated with you today was appropriate.					
Mean scores (SD)		4.31 (0.78)	4.16 (0.90)	4.51 (0.75)	4.63 (0.72)
Frequencies of responses (%)	1	4 (1.0)	8 (1.3)	3 (0.9)	5 (0.9)
	2	10 (2.4)	32 (5.0)	6 (1.8)	10 (1.9)
	3	26 (6.3)	71 (11.1)	16 (4.7)	16 (3.0)
	4	184 (44.9)	268 (41.9)	103 (30.5)	118 (21.9)
	5	186 (45.4)	260 (40.6)	209 (61.8)	391 (72.4)
	Missing	0 (0.0)	1 (0.2)	1 (0.3)	0 (0.0)
6. Overall, you were satisfied with the services you received at this health facility today.					
Mean scores (SD)		4.21 (0.84)	4.13 (0.88)	4.22 (0.85)	4.28 (0.89)
Frequencies of responses (%)	1	4 (1.0)	8 (1.3)	1 (0.3)	5 (0.9)
	2	11 (2.7)	28 (4.4)	13 (3.8)	12 (2.2)
	3	52 (12.7)	78 (12.2)	47 (13.9)	90 (16.7)
	4	172 (42.0)	285 (44.5)	128 (37.9)	150 (27.8)
	5	171 (41.7)	241 (37.7)	149 (44.1)	282 (52.2)
	Missing	0 (0.0)	0 (0.0)	0 (0.0)	1 (0.2)
7. The objective of your visit today was met.					
Mean scores (SD)		4.22 (0.72)	4.16 (0.76)	4.40 (0.84)	4.43 (0.74)
Frequencies of responses (%)	1	4 (1.0)	3 (0.5)	3 (0.9)	6 (1.1)
	2	3 (0.7)	19 (3.0)	13 (3.8)	4 (0.7)
	3	37 (9.0)	67 (10.5)	20 (5.9)	32 (5.9)
	4	220 (53.7)	335 (52.3)	113 (33.4)	205 (38.0)
	5	146 (35.6)	216 (33.8)	189 (55.9)	292 (54.1)
	Missing	0 (0.0)	0 (0.0)	0 (0.0)	1 (0.2)
8. You felt comfortable when you were in the consultation room.					
Mean scores (SD)		3.84 (0.85)	3.93 (0.86)	4.15 (0.82)	4.38 (0.81)
Frequencies of responses (%)	1	5 (1.2)	4 (0.6)	4 (1.2)	5 (0.9)
	2	17 (4.1)	34 (5.3)	9 (2.7)	16 (3.0)
	3	104 (25.4)	136 (21.3)	42 (12.4)	36 (6.7)
	4	197 (48.0)	295 (46.1)	161 (47.6)	197 (36.5)
	5	87 (21.2)	170 (26.6)	122 (36.1)	285 (52.8)
	Missing	0 (0.0)	1 (0.2)	0 (0.0)	1 (0.2)
9. You felt comfortable when you were in the waiting area.					
Mean scores (SD)		3.22 (0.96)	3.42 (1.00)	3.45 (0.94)	3.69 (1.11)
Frequencies of responses (%)	1	26 (6.3)	27 (4.2)	16 (4.7)	32 (5.9)
	2	50 (12.2)	82 (12.8)	36 (10.7)	48 (8.9)
	3	170 (41.5)	204 (31.9)	89 (26.3)	100 (18.5)
	4	136 (33.2)	248 (38.8)	174 (51.5)	229 (42.4)
	5	27 (6.6)	77 (12.0)	22 (6.5)	126 (23.3)
	Missing	1 (0.2)	2 (0.3)	1 (0.3)	5 (0.9)
10. You feel like coming back to this health center if you have the same illness/case in future.					
Mean scores (SD)		4.30 (0.80)	4.25 (0.84)	4.26 (0.78)	4.31 (0.89)
Frequencies of responses (%)	1	4 (1.0)	12 (1.9)	4 (1.2)	16 (3.0)
	2	12 (2.9)	14 (2.2)	7 (2.1)	11 (2.0)
	3	27 (6.6)	53 (8.3)	25 (7.4)	27 (5.0)
	4	183 (44.6)	282 (44.1)	163 (48.2)	220 (40.7)
	5	184 (44.9)	277 (43.3)	137 (40.5)	262 (48.5)
	Missing	0 (0.0)	2 (0.3)	2 (0.6)	4 (0.7)
10-item mean scores (SD)^b^		4.01 (0.51)	3.98 (0.59)	4.07 (0.51)	4.20 (0.59)

a Missing data were imputed by group means to calculate mean scores for individual items.

b Missing data were imputed by individual respondent's means to calculate 10-item mean scores.

Linear regression showed an interaction between intervention (control=0, intervention=1) and data collection stage (first stage=0 and second stage=1) on the client satisfaction scores in the three models (Table 5). Adding fixed effects for facilities to the models did not significantly change the results; therefore, models that were more parsimonious (without the fixed effects) were used in the regression analysis. The first regression model without adjustments indicated that the client satisfaction score increased by 0.06 points in the control group after the intervention, but the difference was not statistically significant. The scores in the intervention group increased by 0.22 points (0.06 [post-intervention stage]+0.16 [interaction effect]), and the increase in the intervention group was significantly more positive than that of the control group (*p=*0.002). The two other models with adjustments for potential confounding factors revealed a significant score increase in the intervention group of 0.20 points (0.07 [post-intervention stage]+0.13 [interaction effect]) in the second model with all of the control variables (*p=*0.015) and 0.19 points (0.06 [post-intervention stage]+0.13 [interaction effect]) in the third model with the control variables selected through backward stepwise regression (*p=*0.014).

**Table 5:** Regression models to measure the intervention's effect on client satisfaction scores (*n=*1,928)

	Model 1			Model 2			Model 3		
			
Variables^a^	Coefficient	SE	*P*	Coefficient	SE	*P*	Coefficient	SE	*P*
Interaction effect (post-intervention stage×intervention facility)	**0.16**	**0.05**	**0.002**	**0.13**	**0.05**	**0.015**	**0.13**	**0.05**	**0.014**
Post-intervention stage	0.06	0.04	0.166	0.07	0.04	0.113	0.06	0.04	0.139
Intervention facility	−0.03	0.04	0.420	−0.00	0.04	0.994	−0.01	0.04	−0.887
Respondent									
Patient^b^	–	–	–	–	–	–	–	–	–
Caretaker	–	–	–	0.04	0.03	0.214	–	–	–
Female sex (respondent)	–	–	–	**0.10**	**0.04**	**0.005**	**0.10**	**0.03**	**0.003**
Age (of respondent)									
18 to <25 years^b^	–	–	–	–	–	–	–	–	–
25 to <35 years	–	–	–	0.01	0.03	0.873	0.02	0.03	0.495
35 to <45 years	–	–	–	0.05	0.04	0.228	**0.08**	**0.04**	**0.041**
≥45 years	–	–	–	0.09	0.05	0.090	**0.13**	**0.05**	**0.005**
Service unit visited									
Maternity	–	–	–	−0.07	0.05	0.159	−0.08	0.05	0.102
Outpatient medical clinic	–	–	–	−0.02	0.05	0.771	−0.03	0.05	0.617
Outpatient nurse clinic^b^	–	–	–	–	–	–	–	–	–
Prenatal care	–	–	–	0.07	0.04	0.119	0.05	0.04	0.252
Inpatient ward	–	–	–	**−0.16**	**0.05**	**0.002**	**−0.15**	**0.05**	**0.002**
Family planning	–	–	–	0.02	0.06	0.749	−0.00	0.06	0.939
Immunization	–	–	–	−0.06	0.05	0.266	−0.04	0.05	0.362
Means of transportation used to visit the facility									
Personal vehicle or motorbike	–	–	–	**0.08**	**0.03**	**0.013**	**0.09**	**0.03**	**0.009**
Public transport	–	–	–	−0.04	0.03	0.279	−0.03	0.03	0.346
By walk or bicycle^b^	–	–	–	–	–	–	–	–	–
Visiting the facility first time	–	–	–	0.01	0.04	0.796	–	–	–
Paid money for the service	–	–	–	**−0.09**	**0.04**	**0.033**	**−0.08**	**0.04**	**0.046**
How long living in the current residence?									
<5 years^b^	–	–	–	–	–	–	–	–	–
5 to <10 years	–	–	–	0.01	0.05	0.812	–	–	–
10 to <20 years	–	–	–	0.02	0.04	0.585	–	–	–
20 to <30 years	–	–	–	0.02	0.04	0.698	–	–	–
≥30 years	–	–	–	0.07	0.04	0.142	–	–	–
Married	–	–	–	0.06	0.04	0.119	0.07	0.04	0.068
Any formal education attended	–	–	–	−0.04	0.03	0.188	–	–	–
Occupation of household head									
Informal sector^b^	–	–	–	–	–	–	–	–	–
Formal sector	–	–	–	**−0.09**	**0.04**	**0.019**	**−0.10**	**0.04**	**0.008**
Working abroad	–	–	–	−0.02	0.05	0.583	−0.02	0.04	0.655
Not working	–	–	–	−0.02	0.08	0.781	−0.02	0.08	0.840
Other	–	–	–	−0.10	0.09	0.269	−0.10	0.09	0.277
Asset index	–	–	–	−0.01	0.01	0.054	**−0.01**	**0.01**	**0.017**
	Adjusted *R*^2^=0.0240			Adjusted *R*^2^ =0.0486			Adjusted *R*^2^=0.0489		

a Among the control variables, the asset index is a continuous variable, while all the others are binomial.

b Reference categories in Model 2 and Model 3.

The bold figures represent statistically significant values (*p*<0.05).

The linear regression also showed that the differences in the client satisfaction scores were significantly associated with the respondents’ characteristics. The backward stepwise regression model indicated significantly higher mean scores among the female respondents (*p=*0.003), clients aged between 35 and 44 years (*p=*0.041), clients who were aged 45 years and older (*p=*0.005), and clients who used a personal vehicle or motorbike to visit the facility (*p=*0.009). Lower client satisfaction scores were associated with visits to the inpatient ward (*p=*0.002), payment of money for the health services (*p=*0.046), the household head working in the formal sector (*p=*0.008), and a higher household possession score (*p=*0.017).

Additional linear regression analyses, conducted separately with the 10 question items, showed the intervention's effect on the scores of each of the 10 Likert-scale question items (Table 6). The score increased significantly for the following question items: ‘The way the healthcare staff communicated with you today was appropriate’ (0.44 points [0.21 for post-intervention stage+0.23 for interaction effect]; *p=*0.002); ‘The explanation about your illness/case and the medication(s) provided by the healthcare staff during the consultation were appropriate’ (0.14 points [−0.09 for post-intervention stage+0.23 for interaction effect]; *p=*0.002); ‘The duration of your consultation by healthcare staff was appropriate’ (0.06 points [−0.13 for post-intervention stage+0.19 for interaction effect]; *p=*0.019); and ‘Overall, you were satisfied with the services you received at this health facility today’ (0.17 points [0.00 for post-intervention stage+0.17 for interaction effect]; *p=*0.044).

**Table 6:** Linear regression analyses of the intervention's effect on responses to each Likert-scale question item (10 separate analyses based on the backward stepwise regression model; *n=*1,928)

	Interaction effect [post-intervention stage×intervention facility]			Post-intervention stage			Intervention facility		
			
Question Items	Coefficient	SE	*P*	Coefficient	SE	*P*	Coefficient	SE	*P*
1. The duration of your consultation by healthcare staff was appropriate.	**0.19**	**0.08**	**0.019**	**−0.13**	**0.06**	**0.048**	−0.01	0.05	0.863
2. The explanation about your illness/case and the medication(s) provided by the healthcare staff during the consultation were appropriate.	**0.23**	**0.08**	**0.002**	−0.09	0.06	0.122	−0.09	0.05	0.081
3. The waiting time before you received the consultation was within a reasonable timeframe.	0.16	0.10	0.119	−0.11	0.08	0.161	−0.04	0.07	0.567
4. The professional competence of the healthcare staff at this health facility is high.	0.11	0.08	0.189	**0.13**	**0.06**	**0.038**	0.02	0.05	0.748
5. The way the healthcare staff communicated with you today was appropriate.	**0.23**	**0.08**	**0.002**	**0.21**	**0.06**	**<0.001**	**−0.12**	**0.05**	**0.021**
6. Overall, you were satisfied with the services you received at this health facility today.	**0.17**	**0.18**	**0.044**	0.00	0.06	0.973	−0.09	0.06	0.090
7. The objective of your visit today was met.	0.09	0.07	0.228	**0.18**	**0.06**	**0.001**	−0.06	0.05	0.196
8. You felt comfortable when you were in the consultation room.	0.10	0.08	0.221	**0.33**	**0.06**	**<0.001**	0.10	0.05	0.059
9. You felt comfortable when you were in the waiting area.	0.04	0.09	0.664	**0.20**	**0.07**	**0.007**	**0.25**	**0.06**	**<0.001**
10. You feel like coming back to this health center if you have the same illness/case in future.	0.09	0.08	0.275	−0.04	0.06	0.541	−0.02	0.05	0.780

The control variables shown in Table 5 were used in the above regression models. The bold figures represent statistically significant values (*p*<0.05).

## Discussion

In this study, several implications were identified regarding the impact of 5S on client satisfaction. First, the 5S intervention appeared to have improved the client satisfaction scores of the patients or caretakers visiting resource-poor health facilities in Senegal. To the best of our knowledge, this is the first quasi-experimental study that showed the impact of 5S in healthcare settings (18). Apart from the qualitative study conducted at the Tambacounda Health Center (17), no other study has highlighted the impact of 5S at resource-poor health facilities in LMICs. Second, the present study's results provided certain ideas about how the 5S intervention led to the increase in client satisfaction scores. Third, our findings filled knowledge gaps about the applicability of 5S in healthcare settings being one of the few empirical studies of a 5S application that highlighted changes using a patient-centeredness measure of healthcare-service quality (18). Additionally, our analyses identified significant associations between the client satisfaction scores and respondents’ demographic and socio-economic characteristics.

This study results illustrate the potential for 5S to improve client satisfaction in resource-poor settings. In Senegal, government health facilities experienced chronic problems related to human resources for health and pharmaceutical supplies (30). Additionally, in the qualitative study conducted at the Tambacounda Health Center, most of the health workers reported poor physical and material resources as obstacles to improving the quality of services (17). It is reasonable to assume that these resource problems were present at all of the health centers in our study. However, the 5S does not directly address resource problems. In addition, the noted positive impact under the intervention program in two regions implies the applicability of 5S as part of a broader strategic framework of healthcare-service quality improvement in LMICs.

Our results also provide information about changes in client satisfaction. In the linear regression analyses of the individual Likert items, the client satisfaction score increased significantly in four items, of which three pertain to the healthcare staff members’ attitudes (i.e. their communication, their explanations about the patient's illness or case and medications, and the duration of their consultations). The impact on the healthcare staff members’ attitudes identified in this study reinforces the findings of the qualitative study conducted at the Tambacounda Health Center (17), in which 5S was considered to have the potential to motivate health workers. However, the study's results could not explain whether the attitude change resulted from the improved work environment due to 5S, or the staff members’ experience of participating in the 5S intervention process itself. To explore more effective intervention modalities, further studies need to address the mechanisms by which 5S leads to attitude changes in healthcare staff.

The changes in client satisfaction identified in this study meet knowledge gaps about the applicability of 5S in improving healthcare-service quality. Several previous studies highlighted changes in service quality resulting from the 5S intervention. However, all such studies compared the facility's status before and after the intervention without adopting explicit measures to control for potential confounding. Based on the classification of the healthcare quality dimensions proposed by the Institute of Medicine (US) (31), changes in efficiency measures reported in other studies include improvements in work processes, potential cost reductions, and increases in physical space (11, 13–15). Changes in safety measures, such as a reduction in the nosocomial infection rate, also have been described in other studies (10, 16). However, client satisfaction (the focus here) is not commonly studied as a patient-centeredness measure of healthcare-service quality. In addition, our study showed the potential for 5S to improve this aspect of healthcare-service quality in resource-poor government health facilities that provide primary care.

Moreover, according to the linear regression analysis, client satisfaction was significantly associated with several control variables that represented the respondents’ demographic and socio-economic characteristics. The satisfaction level was affected by the respondents’ gender, age, the household head's occupation, and the household's possessions. The logic underlying these associations cannot be determined from our study's results. This may be attributed to the different respondent groups’ perceptions of the services, or their different experience because of the diverse attitudes of the healthcare staff members based on the respondents’ demographic and socio-economic characteristics. Although these findings were not the focus of this study, they highlighted areas requiring further research. Several studies have investigated the association between client satisfaction and factors related to healthcare-service provision, such as service quality and patients’ experience (32, 33); however, little research has been conducted on demographic and socio-economic characteristics as determinants of client satisfaction in LMICs.

This study has several limitations. First, the facilities could not be randomized because of the deferred transfer of several health centers to new facilities. Nevertheless, our results indicated that this constraint did not affect the measured impact of the intervention. Second, it was impossible to regulate information sharing pertaining to 5S among the staff members of the control and intervention facilities because of periodic gatherings for events, such as quarterly coordination meetings conducted at the regional level. Third, considering previous findings of the qualitative study conducted at Tambacounda Health Center (17), the idea of including a measure to assess health workers’ attitudes was initially envisaged in the study's design. However, it was difficult to control potential bias toward health workers’ positive responses at the post-intervention stage because of their direct involvement in the 5S intervention process. Therefore, the target population in this study was limited to patients and caretakers whose answers were less likely to be biased by the intervention.

## Conclusions

There were significant improvements in client satisfaction scores at government health centers in two regions of Senegal after the intervention. Thus, 5S has the potential to improve client satisfaction in resource-poor health facilities although it does not directly address resource problems. The 5S could therefore be recommended as a strategic option for healthcare-service quality improvement in LMICs. In addition, patients or caretakers perceived improved attitudes in healthcare staff members following the 5S intervention. To explore more effective intervention modalities, further studies need to address the mechanisms by which 5S leads to attitude changes in healthcare staff.

## Paper context

The 5S has been introduced in healthcare facilities in high-, low-, and middle-income countries; however, evidence of its impact is limited. This study contributes to the growing body of evidence on the impact of the 5S intervention, particularly with regard to improving client satisfaction in resource-poor health facilities. In order to explore more effective intervention modalities, further studies are necessary to better understand the mechanisms by which the 5S can improve client satisfaction.
